# Device errors in asthma and COPD: systematic literature review and meta-analysis

**DOI:** 10.1038/s41533-017-0016-z

**Published:** 2017-04-03

**Authors:** Henry Chrystyn, Job van der Palen, Raj Sharma, Neil Barnes, Bruno Delafont, Anadi Mahajan, Mike Thomas

**Affiliations:** 1 Inhalation Consultancy Ltd, Yeadon, Leeds and Faculty of Human and Health Sciences, Plymouth University, Plymouth, UK; 2 Department of Research Methodology, Measurement, and Data Analysis, Faculty of Behavioural, Management and Social Sciences, University of Twente, Enschede, The Netherlands; 3 Department of Epidemiology, Medisch Spectrum Twente, Enschede, The Netherlands; 4 GSK, Brentford, UK; 5 William Harvey Institute, St Bartholomew Hospital and The London School of Medicine and Dentistry, London, UK; 6 Exploristics Ltd, Belfast, UK; 7 Bridge Medical, Richmond, UK; 8 Primary Care and Population Sciences, University of Southampton, Southampton, UK; 9 NIHR Southampton Respiratory Biomedical Research Unit, Southampton, UK; 10 NIHR Wessex Collaboration for Leadership in Applied Health Research and Care (CLAHRC), Southampton, UK

## Abstract

Inhaler device errors are common and may impact the effectiveness of the delivered drug. There is a paucity of up-to-date systematic reviews (SRs) or meta-analyses (MAs) of device errors in asthma and chronic obstructive pulmonary disease (COPD) patients. This SR and MA provides an estimate of overall error rates (both critical and non-critical) by device type and evaluates factors associated with inhaler misuse. The following databases from inception to July 23, 2014 (Embase®, MEDLINE®, MEDLINE® In-Process and CENTRAL) were searched, using predefined search terms. Studies in adult males and females with asthma or COPD, reporting at least one overall or critical error, using metered dose inhalers and dry powder inhalers were included. Random-effect MAs were performed to estimate device error rates and to compare pairs of devices. Overall and critical error rates were high across all devices, ranging from 50–100% and 14–92%, respectively. However, between-study heterogeneity was also generally >90% (I-squared statistic), indicating large variability between studies. A trend towards higher error rates with assessments comprising a larger number of steps was observed; however no consistent pattern was identified. This SR and MA highlights the relatively limited body of evidence assessing device errors and the lack of standardised checklists. There is currently insufficient evidence to determine differences in error rates between different inhaler devices and their impact on clinical outcomes. A key step in improving our knowledge on this topic would be the development of standardised checklists for each device.

## Chronic lung diseases: Call to standardise research into inhaler device errors

Researchers should adopt a standardised approach to investigate the incorrect use of inhalers and its associated clinical implications. Henry Chrystyn at Plymouth University, together with scientists across the UK and the Netherlands, conducted a review of research related to inhaled medication errors made by patients with asthma or chronic obstructive pulmonary disease. It is widely acknowledged that many patients with lung conditions don’t use their inhaler devices correctly, which affects drug effectiveness and disease control. While Chrystyn’s team found high critical error rates reported across all devices, their meta-analysis and systematic review highlighted significant gaps in knowledge regarding different inhalers and associated error rates, and how these affect clinical outcomes. The researchers call for in-depth studies into device use, alongside standardised checklists and definitions for such studies to use to ensure consistency.

## Introduction

Inhaled medications are fundamental to the treatment of asthma and chronic obstructive pulmonary disease (COPD), with inhaler devices being the principal route for administering such treatments.^1, 2^ Many different types of inhaler are available, but pressurized metered dose inhalers (pMDIs) and dry powder inhalers (DPIs) are the devices most commonly used for drug delivery in the treatment of asthma and COPD.^1, 2^ A large number of asthma and COPD patients do not use their inhaler devices correctly. Errors in device use may impact the effectiveness of the delivered drug and thereby lead to the sub-optimal control of asthma and COPD.^3–6^ It is therefore important to understand and quantify device-use errors so that patient interventions can be effectively introduced and new devices designed to avoid common errors.

The literature highlights the fact that the definitions of critical and non-critical errors, as well as the number and type of checklist steps, vary widely between different devices and studies. A critical error is one that may impact the effectiveness of the delivered drug and thereby lead to the sub-optimal disease control of asthma and COPD,^3, 7^ whereas a non-critical error is one of the checklist steps for a particular device that is not classified as critical.

A previous systematic review (SR) focused on errors with DPI devices only^7^ and another has found that there has been no change in the type and number of errors reported over the past 40 years.^8^ The present SR and MA was conducted to provide an estimate of error rates (i.e., the proportion of patients with at least one error, critical and/or non-critical) by device type and to evaluate the factors associated with inhaler misuse, for example device and patient characteristics. In addition, the use of educational interventions designed to improve inhaler technique was investigated.

## Results

### Search results

**Fig. 1:**
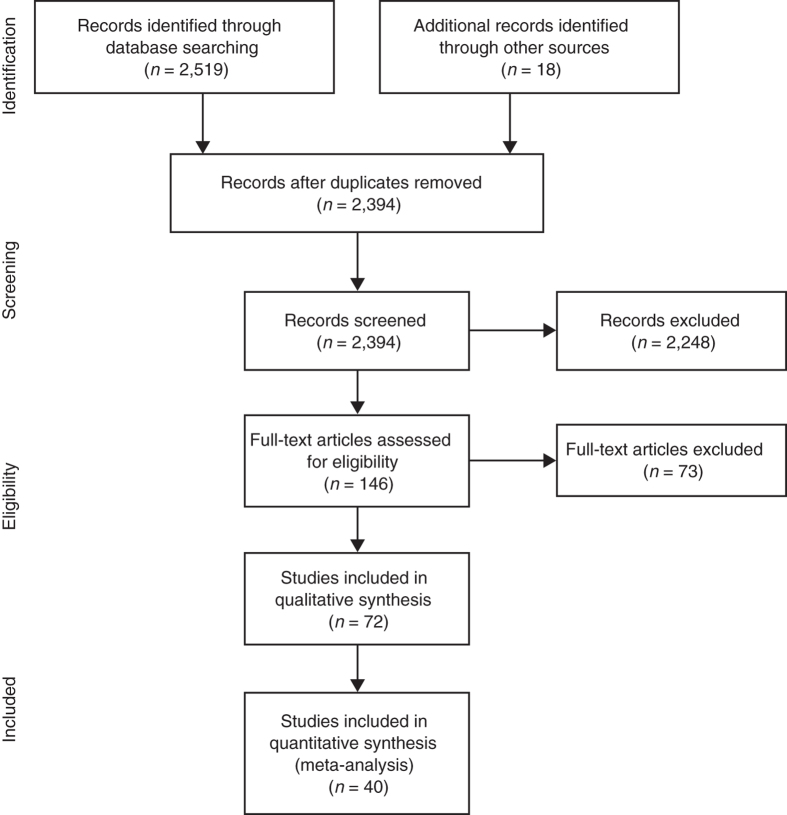
PRISMA flow diagram describing the results of the systematic review

The search results for the SR are shown in the PRISMA flow diagram (Fig. 1). Overall, a total of 2519 citations were identified via database searching and a further 18 were identified through back-referencing of reviews and other relevant primary studies. After screening, 72 primary studies were extracted, all of which were included in the SR and 40 of these were selected for inclusion in the MA, based on the predefined criteria previously described (Fig. 1). Reasons for study exclusion are provided as Supplemental data (Appendix 1).

### Study characteristics

The majority (54%) of the 72 identified studies included in the SR comprised patients with both asthma and COPD, while 32% and 14% were conducted in either asthma or COPD patients, respectively. Most (90%) of the mixed patient studies did not report data for the asthma or COPD subgroups separately.

Fifty-four observational studies were retrieved. Of these, 40 were of a cross-sectional design and the remaining studies were prospective. Only 18 randomised controlled trials (RCTs) were identified, of which 11 were crossover studies and the other seven were of a parallel group design. Approximately 80% of the observational studies included in the MAs were conducted in an outpatient population and with patients primarily utilising their existing inhaler device. Baseline data were obtained prior to device training for most of the RCTs.

The majority of extracted studies evaluated MDI (*n* = 29) or DPI (*n* = 32) inhalers and reported either the proportion of patients with any error (critical plus non-critical) or those with any critical error. Amongst the DPI studies, most involved Turbuhaler® (*n* = 17) or Diskus® (*n* = 15) devices. Fifty-four studies were conducted in patients who were regular inhaler users and assessed the inhalation technique that patients had been employing on a regular basis.

Due to limited data availability, MAs of any device comparisons were not feasible for the: i) overall error frequency (cross-sectional studies and RCTs) and ii) critical error rates for the RCTs.

Most of the studies included in the overall analysis (*n* = 72) were conducted in the USA (*n* = 13), the Netherlands (*n* = 10) and the UK (*n* = 10).

Amongst all of the studies, about two-thirds reported that the assessor was trained in the inhalation technique of the device under assessment. The assessors included pharmacists, physicians/general practitioners (GPs)/specialists, students, investigators, research assistants and technicians. In the majority (>90%) of cases, inhalation technique was assessed utilising author-validated, existing checklists. Furthermore, a variety of checklists was used for the same device across different studies. These checklists were highly variable and differed in both the number of steps and their definition(s) of these steps. Errors arose from failure to correctly complete the relevant checklist steps for a particular device.

Supplementary Table 2 details the characteristics of all studies that reported overall and critical error rates. The number of studies included in the MA was lower (<72) because studies that reported, i) an error frequency using a definition other than that previously defined, ii) pooled data, or iii) error rates for each individual step, but not cumulatively, were excluded.

### Overall error rate (critical and non-critical)

**Fig. 2:**
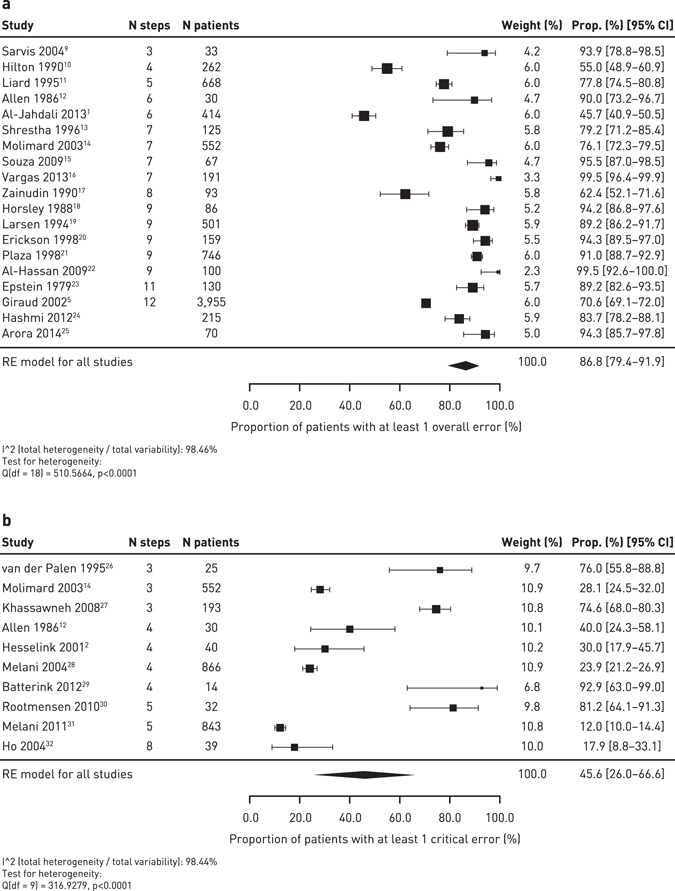
Meta-analysis of the overall error rate frequency (**a**) and critical error rate frequency (**b**) for MDIs in prospective/cross-sectional studies

**Fig. 3:**
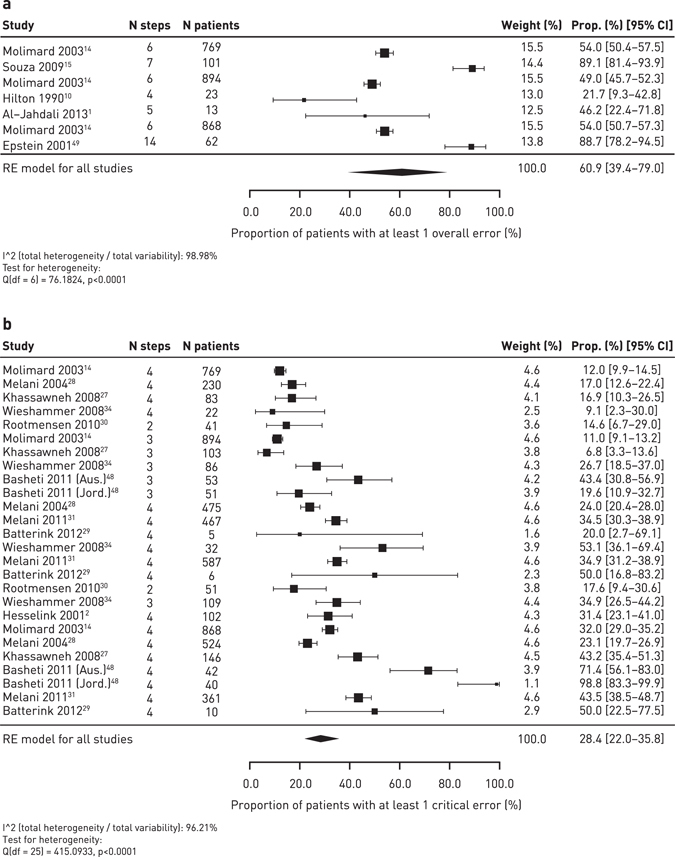
Meta-analysis of the overall error rate frequency (**a**) and critical error frequency (**b**) for DPIs in prospective/cross-sectional studies

Across the devices, error rates appeared common with approximately 50–100% of patients experiencing at least one error. The pooled summary results for the MDI devices estimated an overall error frequency (RE model for all studies) of 86.8% [95% CI 79.4–91.9] of patients with at least one error (Fig. 2a). However, there was a high level of heterogeneity between the studies (98.5% I-squared statistic). Compared with MDIs, relatively few studies assessed the overall error rates for DPI devices. The pooled summary results for DPIs estimated an overall error frequency (RE model for all studies) of 60.9% [95% CI 39.4–79.0] of patients with at least one error, with a high level of between-study heterogeneity (99.0% I-squared statistic; Fig. 3a).

The frequency of overall error rates for individual devices is shown in Table 1. None of the studies assessed the overall or critical error rates for the Breezhaler®, Easyhaler®, Ellipta®, Elpenhaler®, Genuair®, Nexthaler®, or Novolizer® devices; devices with zero studies have been excluded from the table. An MA of overall error frequency for Turbuhaler® and Diskus® in prospective and cross-sectional studies is shown as Supplemental data (Appendix 2, Supplementary Figure 1). The most common overall errors by device are detailed in Table 2. A sensitivity analysis of the overall error frequency for MDIs, conducted to assess any bias due to the inclusion of industry-sponsored studies, gave a similar finding (87.6% [95% CI 79.4–92.9]; RE model for all studies) to the pooled summary (Appendix 3; Supplementary Figure 3).

**Table 1:** Frequency of overall error with devices (quantitative and qualitative estimates)

Device	Cross-sectional studies			RCTs		
	Number of studies	Pooled estimate [95% CI] (I-squared)^*^	Range of error frequency	Number of studies	Pooled estimate [95% CI] (I-squared)*	Range of error frequency
MDI	19	86.8% [79.4–91.95] (98.5%)	45.7–100.0%	2	89.5% [75.7–95.9] (60.0%)	85.1–94.2%
MDI with spacer	3	52.0% [24.3–78.6] (87.3%)	31.6–78.0%	0	N/A^**‡**^	N/A^**‡**^
BA-MDI (Autohaler^®^)	2	57.7% [52.9–62.5] (84.9%)	54.9–59.9%	0	N/A^**‡**^	N/A^**‡**^
Turbuhaler^®^	4	55.5% [24.5–82.7] (93.6%)	22.0–88.7%	3	73.6% [55.6–86.1] (92.1%)	54.0–82.2%
Diskus^®^	1	N/A^**‡**^	49.0%	3	62.4% [51.9–72.0] (61.6%)	50.0–68.3%
Aerolizer^®^	2	75.1% [31.0–95.3] (97.2%)	54.0–89.1%	0	N/A^**‡**^	N/A^**‡**^
Handihaler^®^	0	N/A^**‡**^	N/A^**‡**^	1	N/A^**‡**^	66.7%

*N/A* Not applicable

* I-squared statistic indicates the percentage of variance that is attributable to between-study heterogeneity. The higher the percentage, the greater the heterogeneity. ^‡^Not applicable as number of studies was ≤1

**Table 2:** Most common overall errors reported for each device

Device	No exhalation before inhalation (% frequency—range)	Number of studies	Not holding breath for a few secs after inhalation (% frequency—range)	Number of studies	Not using a proper seal around mouthpiece (% frequency—range)	Number of studies
MDI	10.2–60.2	14	46.7–76.7	18		
MDI with spacer	12.1–73.9	2	22.8–79.7	3	0–28.0	5
BA-MDI (Autohaler^®^)	22.3–23.0	2	30.2–38.7	2		
Turbuhaler^®^	16.0–75.0	12	6.0–77.1	15	0–21.7	5
Diskus^®^	20.6–65.8	9		12	0–15.1	10
Aerolizer^®^	24.5–77.1	4	18.7–77.1	5		
Handihaler^®^	25.0–77.1	3	24.7–77.1	4		

### Critical error rates

Across the devices, critical error rates appeared common with approximately 14–92% of patients experiencing at least one critical error. The frequency of critical error rates for individual devices is shown in Table 3. The pooled summary results for MDIs estimated a critical error frequency of 45.6% [95% CI 26.0–66.6] of patients with at least one critical error (*n* = 10 studies), however, the data were highly heterogeneous (98.4% I-squared statistic) (Fig. 2b). The critical error rates for the DPIs were highly variable for each device: Aerolizer® (*n* = 4 studies) 14.2% [95% CI 11.0–18.1], Diskus® (*n* = 9 studies) 20.8% [95% CI 13.7–30.2], Turbuhaler® (*n* = 10 studies) 40.1% [95% CI 28.6–52.9], and Handihaler® (*n* = 3 studies) 42.4% [95% CI 28.8–57.1]. The pooled summary results for DPIs estimated a frequency of 28.4% [95% CI 22.0–35.8] of patients with at least one critical error (96.2% I-squared statistic; Fig. 3b). The between-study variability was high (>90% I-squared statistic) for the Diskus® and Turbuhaler® devices. The heterogeneity was lower for the Aerolizer® (44.3% I-squared statistic) and Handihaler® (58.4% I-squared statistic) devices but there were fewer studies available for inclusion, so the between-study variability may be under-estimated.

**Table 3:** Frequency of critical error with devices (quantitative and qualitative estimates)

Device	Cross-sectional studies			RCTs		
	Number of studies	Pooled estimate [95% CI] (I-squared)*	Range of error frequency	Number of studies	Pooled estimate [95% CI] (I-squared)*	Range of error frequency
MDI	10	45.6% [26.0–66.6] (98.4%)	12.0–93.0%	0	N/A^‡^	N/A^‡^
MDI with spacer	3	8.9% [0.9–50.5] (93.7%)	2.6–47.0%	0	N/A^‡^	N/A^‡^
BA-MDI (Autohaler^®^)	1	N/A^‡^	11.0%	0	N/A^‡^	N/A^‡^
Turbuhaler^®^	10	40.1% [28.6–52.9] (95.7%)	18.0–100%	1	N/A^**‡**^	26.0%
Diskus^®^	9	20.8% [13.7–30.2] (93.6%)	6.8–43.0%	3	18.0% [8.2–35.1] (84.2%)	8.0–31.9%
Aerolizer^®^	4	14.2% [11.0–18.1] (44.3%)	9.1–16.8%	0	N/A^‡^	N/A^‡^
Handihaler^®^	3	42.4% [28.8–57.1] (58.4%)	35.0–53.1%	0	N/A^‡^	N/A^‡^

*N/A* Not applicable

^*^ I-squared statistic indicates the percentage of variance that is attributable to between-study heterogeneity. The higher the percentage, the greater the heterogeneity. ^‡^Not applicable as number of studies was ≤1

An MA of critical error frequency for Turbuhaler® and Diskus® in prospective and cross-sectional studies is shown as Supplemental data (Appendix 2, Supplementary Figure 2). MA using the RCTs was only feasible for the Diskus® inhaler. The pooled critical error estimate was 18.0% [95% CI 8.2–35.1] and the between-study heterogeneity was relatively high (84.2% I-squared statistic).

There were insufficient studies available (≤1) to enable quantitative analysis for the Autohaler®, Breezhaler®, Easyhaler®, Ellipta®, Elpenhaler®, Genuair®, Nexthaler® and Novolizer® devices.

### Differences in error rates between devices

There was only sufficient evidence to conduct MAs of the critical error frequency for the observational studies.

### Critical errors

The only significant results were for the comparison between the Diskus® and Turbuhaler® devices, odds ratio (OR): 2.90 [95% CI 1.41–5.96] (*n* = 9 studies) indicating fewer errors when using the Diskus® device, although the heterogeneity between studies was still very high (93.9% I-squared statistic). The pooled OR for critical error frequency favoured Turbuhaler® over MDI (OR: 1.76 [95% CI 0.53–5.85], *n* = 7 studies; 93.9% I-squared statistic) and Diskus® vs. MDI (OR: 5.01 [95% CI 0.87–28.69], *n* = 6 studies). However, in both cases, the heterogeneity between studies was >98% (I-squared statistic).

Trend differences were observed for the following device comparisons for critical errors: Turbuhaler® vs. MDI (OR: 1.76 [95% CI 0.53–5.85]), Diskus® vs. MDI (OR: 5.01 [95% CI 0.87–28.69]), and Turbuhaler® vs. Handihaler® (OR: 1.09 [95% CI 0.47–2.53]), but the results were non-significant.^3, 11, 16, 18, 25, 28, 30, 31, 33–39^

A sensitivity analysis, conducted to assess any bias due to the inclusion of industry-sponsored studies, provided similar results (Appendix 3; Supplementary Figures 4–6).

### Impact of patient and study characteristics

The meta-regression analysis showed no significant findings for different baseline characteristics. However, a qualitative review of the extracted studies that analysed data according to, i) patient, ii) disease, and iii) other characteristics, and assessed the association of these factors with the likelihood of making a device error was conducted. A total of 37 primary studies assessed predictors of inhalation technique errors.

Common patient characteristics that were reported to impact device error rates included: i) age (*n* = 15 studies, with patients in older age groups reporting more errors compared with younger patients);^3, 11, 16, 18, 25, 28, 30, 31, 33–39^ ii) education (*n* = 9 studies)^11, 16, 22, 24, 28, 31, 35, 36, 40^ with a higher level of education being associated with fewer errors; iii) COPD diagnosis (*n* = 6 studies);^15, 23, 27, 28, 31, 41^ iv) gender (females reporting a higher error frequency);^28, 35, 42^ v) socioeconomic status (low income was associated with a higher frequency of errors); and vi) relationship status, number of comorbidities and disease severity (*n* = 1 study each).^34, 43^

In studies that recruited patients with asthma or COPD, there was a higher error frequency in patients with COPD (*n* = 6 studies).^15, 23, 27, 28, 31, 41^ In the four studies that included only COPD patients,^15, 22, 26, 29^ two reported the overall error frequency (>93% of patients had at least one error,^15, 22^ MDI and Aerolizer® devices). In the two studies reporting critical error frequency,^26, 29^ 93% and 76% of patients were reported to have at least one critical error, respectively. However, the sample size was relatively small (*n* < 30) in three of the studies. Of the eight studies that recruited only asthma patients,^3, 4, 10, 11, 13, 17, 23, 24^ four reported an overall error frequency of <75%. Critical error frequency was not reported.

Inhaler-related characteristics reported to impact device error rates included prior training on device use,^3, 11, 26, 28, 30, 31, 34, 36, 40, 41^ and duration of device use and use of multiple inhalers (*n* = 3 studies each).^25, 29, 41^ Receipt of prior training on device use was predictive of a lower error frequency compared with no prior training; further, a longer duration of device training compared with shorter duration of training, and receipt of a practical demonstration of correct inhalation technique were factors also associated with lower error frequency rates. A longer duration of device use was predictive of a higher error rate, compared with those who had received their inhaler devices more recently. Use of multiple inhalers also predicted a higher error frequency.

Other characteristics that were reported to affect device error frequency included regular clinic visits,^4, 28^ polypharmacy,^18, 41^ and uncontrolled disease^4, 31^ (*n* = 2 studies each). No trend was observed with regards to patient setting in either overall or critical error frequency.

There was a large variability in the sample size of the included studies. In general, studies with a smaller sample size reported a higher frequency of device errors, particularly critical errors.^26, 29, 30^ There was also a large variation in the number of steps assessed for the different inhaler devices, with studies including between three steps^9^ and 12 steps^5^ for overall error assessment. The relationship between number of steps and error rate was investigated by ordering forest plots of error rates by the number of steps in each study. There was a trend towards higher error rates with a larger number of steps, however no consistent pattern could be observed.

## Discussion

No SR or MA of this type has been previously published. The SR conducted by Lavorini and colleagues^7^ was limited as it focused on DPI devices only and an MA of device errors was not undertaken. The present SR of the existing data provides a valuable and timely assessment of the quality of the existing evidence base surrounding device errors. Despite limitations of the data, it can be seen that both the overall and critical error rates are reported to be high across all devices, ranging from 50–100% and 14–92%, respectively. Although there were very limited data on error rates and symptom control/long-term outcomes, one might hypothesise that correct use of the device is fundamental to the efficacy of the drug, and in this case, the reported error rates and critical error rates may result in sub-optimal treatment and disease control.

There were insufficient high-quality data to draw definitive conclusions about the comparative error frequency between different devices. Meta-regression analyses of patient and study characteristics were inconclusive. However, previous studies have reported associations between certain patient and study characteristics and device error rates (although these data were not quantified). Some studies have reported that older age^31, 33, 35^ and female gender^35, 44^ are associated with higher device error rates. Other socio-economic factors have previously been reported to influence device error frequency. A higher level of education has been reported to be associated with a lower frequency of errors.^25, 31, 36^ Additionally, a higher frequency of errors was found in patients with COPD than those with asthma.^15, 28^ Other factors reported in the literature to impact error frequency were receipt of prior training and type of training,^36^ duration of device use^29^ and use of multiple inhalers.^27, 30^ This may be because patients with a longer duration of device use are likely to have only received training/instruction when the device was first prescribed. The higher error rate reported with multiple inhaler usage may have been due to the higher burden and confusion associated with the use of different devices.

### Limitations of the available data

Given the importance of this area of research, the number of publications focusing on device errors is relatively low, especially when compared with the overall volume of clinical research publications in COPD and asthma. Additionally, there was very little information on the association between device error rates and clinical outcomes. From the available publications, the overall quality was moderate for the majority of studies, with few high-quality studies^14, 27, 30, 31, 33^ (Supplementary Table 2). Whilst it is possible to draw qualitative learning from across the studies, the lack of consistency in studying device errors means that the MAs have to be interpreted with caution.

There were several potential sources of heterogeneity between the different studies including: i) differences in disease diagnosis; ii) heterogeneity due to varying study types; iii) large variability in the level of training received by patients; iv) variability in device step checklists (one of the most important limitations); v) variability in assessors’ technique; vi) the subjectivity of each assessment; vii) heterogeneity due to patient-introduced bias (the Hawthorne effect); viii) studies not specifically designed to assess error rates between devices. This inter-study heterogeneity and lack of consensus around error rates and the types of errors associated with different devices may have an impact in the clinic, with healthcare professionals unclear about which inhaler to prescribe for their patients.

In terms of diagnosis, the majority of studies recruited both asthma and COPD patients and did not provide information on the sub-groups according to disease type.^25, 27^ Moreover, there was no validation of the disease diagnoses reported in the papers. Amongst the cross-sectional studies, patients were generally observed using their regular inhaler.^14, 31^ However, in the RCTs, patients were assessed in a controlled manner and included normally recruited device-naïve participants only.^45, 46^

In the cross-sectional studies, device technique was assessed in patients without any study protocol-specific training or instruction prior to study entry. Moreover, the majority of the studies included in the MA specified a “lack of training regarding device use” as the primary reason for device mishandling.^14, 31^ Patients who received training had usually received it at the time of first prescription of the device and did not receive any additional follow-up training or further assessment of their inhaler technique.^31^ Additionally, the medical personnel responsible for teaching the correct inhaler technique were reported to be lacking in basic training.^6^ Inhalation technique was assessed by a variety of assessors including trained pharmacists,^22, 27^ respiratory specialists,^40^ GPs,^3, 14^ or others (including trained assistants to the physician, intern students, or laboratory staff). Additionally, not all of the studies provided information regarding the assessors.

There was also variability in the number of steps, the actual details of the device checklist steps and in the definitions of critical and non-critical errors. For example, for the Diskus® device, the number of overall and critical errors possible varied between 5–12 and 2–4, respectively^29–31, 47, 48^ and the frequency of errors for the Turbuhaler® was 4–14 and 3–5, respectively.^2, 10, 30, 49^ There was a trend towards higher error rates with a larger number of steps, however no consistent pattern was observed. The definition of critical errors used in the majority (approximately 90%) of studies (e.g., “the proportion of patients with an error for a step that is deemed necessary for the adequate delivery of the drug to the lungs”) is also highly ambiguous (i.e., adequate could mean anywhere between 20–100% drug delivery).

There may also be heterogeneity due to patient-introduced bias, i.e., the Hawthorne effect. This is when the patient is being observed for the technique as part of the study/studies where there is a high probability that the patient will try to use the inhaler in the best possible way.^37^ This may not result in a true reflection of their daily use and leads to an under-estimation of the error frequency. A few studies specified that inhaler technique demonstration took place in an empty room and that the videotaped observations were assessed by nurses.^50^

Finally, although the quality of the data included in these studies was good (Supplementary Table 2), the studies included in these comparisons were largely cross-sectional or prospective in nature and were not designed to compare error rates between different devices. Utilising them in this way necessitates caution. Additionally, a number of the studies had a relatively small sample size, for example, the Batterink study^29^ showed significant differences in error rates for comparisons between MDIs and Turbuhaler® and Diskus® devices, but only included ten and five patients, respectively for the latter two devices.

### Limitations of the review methodology

There are a number of limitations associated with this MA. Firstly, studies that reported error frequency using a definition other than that previously defined were not included in the analysis. This excluded approximately 10% of the studies. Secondly, studies that reported error rates for each individual step, but not cumulatively, were described qualitatively only (approximately 10% of studies). Additionally, studies that reported pooled data, i.e., reported for all DPI devices, were not included in the analysis (*n* = 3 studies).

### Main implications for clinical practice and research

#### Clinical research

There is a need to standardise the definitions of non-critical and critical device errors and their assessment, as well as improve clarity on the clinical importance of each error. Indeed, the literature highlights that definitions of critical and non-critical errors can vary substantially between different studies. It is essential that the wider scientific community reaches a consensus regarding error terminology for the different inhaler devices and develops a standardised checklist for each device, similar to that which is available in the Netherlands.^51^ This will not only be useful for standardising the conduct of future clinical research but also will also provide a valuable clinical tool and enable comparison of devices across future studies.

It should be noted that a number of errors are common to both the MDI and DPI devices, e.g., “exhaling before inhalation” and “holding breath for a few seconds after inhalation”. “Ensuring a proper seal around mouthpiece” is common for MDI with spacer, Diskus® and Turbuhaler® devices (Table 2). It was not possible to identify common errors for Autohaler®, Breezhaler®, Elpenhaler®, Genuair®, Nexthaler® or Novolizer® due to limited data. These steps may provide the basis for the identification and refinement of errors. However, there appears to be more inconsistency in the definition of critical errors.

Once the step checklists and critical/non-critical errors have been standardised, there is a need to conduct more systematic clinical research in this area. There is currently insufficient evidence to be able to determine whether there are any differences in error (non-critical and critical) rates between different inhaler devices. Prospective clinical studies in inhaler-naïve patients, using more objective device training, are required in order to address this issue.

During routine clinical practice, inhaler technique errors may be compensated by increasing the medication dose if disease control is sub-optimal, but this has not yet been systematically studied. Conducting a study to assess low medication doses where the impact of critical device errors is likely to be more pronounced would provide a useful approach to assess the impact of various inhalation steps and errors on patient outcomes. Another approach would be to assess treatments that are available across a number of different devices at microgram-equivalent doses, and to conduct real-world studies in COPD and asthma patients (individually and combined).

Finally, as the outcomes data are very limited, there is a need to investigate the links between different critical device errors and long-term, clinical effectiveness (patient outcomes), resource use and adherence rates.

#### Clinical practice

There has been awareness of the problem of device errors for over 40 years^8^ but this issue has still not been resolved. The high error rates observed across inhalation devices in this study suggests that more time should be invested by healthcare professionals in educating/training patients on how to operate their inhalers correctly. There are several factors that need to be addressed, including the requirement for standardised training of healthcare professionals and patients for the different inhaler devices and regular re-evaluation of inhaler technique and mastery. Existing guidelines^3, 4, 49^ provide targets for device training but these have still not been achieved. The development of standardised training protocols and schedules for the individual inhaler devices may aid this process. There is also a need to assess the ease of training and continued mastery across the different devices.

Once sound research techniques are available, this could lead to improvements in clinical practice whereby training is standardised and conducted on an ongoing basis, with regular re-assessment of patient device handling.

## Conclusions

This SR and MA highlight the relatively limited body of evidence assessing device errors that is currently available, given the importance of this issue. From the available data in the literature, it is apparent that patients are not operating their inhalers correctly. Overall and critical error rates appear to be high across all of the devices assessed: approximately 50–100% and 14–92%, respectively. However, the high level of heterogeneity between studies prevents any definitive conclusions being drawn. There is currently insufficient evidence to be able to determine whether there are any differences in error (non-critical and critical) rates between different inhaler devices. Furthermore, there are limited available data assessing the impact of device errors on clinical outcomes. There is a need to develop and utilise consistent definitions of non-critical and critical device errors and to develop standardised checklists for each individual device in order to facilitate future clinical research and enable comparability between studies. The development of standardised training protocols for the individual inhaler devices will also aid this process.

## Methods

### Search strategy

Excerpta Medica Database [Embase®], Medical Literature Analysis and Retrieval System Online [MEDLINE®], MEDLINE® In-Process (to ensure that non-indexed citations were retrieved) and the Cochrane central register of controlled trials (CENTRAL) databases were searched from inception to July 23, 2014. MEDLINE® and Embase® were searched using embase.com and CENTRAL and MEDLINE® In-Process were searched using Cochrane library and  Pubmed.com interfaces, respectively. The search strategy is summarised in Supplementary Table 1.

### Inclusion/exclusion criteria

Studies in adult males and females with asthma or COPD (all severity grades) were included in the analysis. The devices of interest were MDIs (both with and without spacers; chlorofluorocarbon/hydrofluoroalkane [CFC/HFA] propellant; breath-actuated [Autohaler® and EasiBreathe®] and Respimat® [soft mist]) and DPIs (Aerolizer®, Breezhaler®, Diskus®/Accuhaler®, Easyhaler®, Ellipta®, Elpenhaler®, Genuair®, Handihaler®, Nexthaler®, Novolizer® and Turbuhaler®).

### Data extraction

All citations (titles and abstracts) were screened for eligibility against the pre-specified inclusion/exclusion criteria. Full publications of the included citations were then reviewed for eligibility. All the citations excluded at the title/abstract or full-text screening stage were coded and the reasons for exclusion recorded.

### Systematic review

Studies identified from the full-text screening stage that evaluated the number of patients with at least one overall error (critical plus non-critical) or at least one critical error, or assessed error frequency at each step for a specific device, were included in the SR.

### Meta-analyses

Studies reporting at least one overall or critical error were included in the MA. For RCTs and prospective observational studies, the baseline error frequency prior to any study-related training or instruction was included in the analyses. Some of the device data were excluded from the MA because they did not meet criteria for the minimum number of studies and/or patients.

### Quality assessment

The quality of all the included studies was assessed. The quality of RCTs was determined using the criteria published by the Cochrane Collaboration, with respect to different types of bias, and the quality of cross-sectional and observational studies was evaluated using the relevant Newcastle and Ottawa scale [2008]. Decisions about the estimated risk of bias were used to help evaluate heterogeneity between the studies.

### Statistical analysis

Data from cross-sectional studies and RCTs were analysed separately.

For all quantitative analyses, data were evaluated for the proportion of patients with at least one overall error and those with at least one critical error. Studies that reported error frequency using a definition other than that previously defined and studies that reported pooled data, i.e., reported for all DPI devices, were not included in the analysis. Additionally, studies that reported error rates for each individual step, but not cumulatively, were described only qualitatively. Data were excluded from the analysis if there were fewer than five patients using a particular device within a study. MDI devices were categorised into two different sub-groups: i) MDI alone, ii) MDI with spacer.

A random-effects model was used for all the MAs in order to account for between-study heterogeneity.

### Meta-analyses of device error frequency for each device/device type

All studies selected for analysis, and providing data for an individual device, were included in the MAs. The overall device error frequency was summarised for each device/device type using a restricted maximum likelihood (REML) random-effects MA. These analyses were performed when at least two studies provided adequate data for the same device. It should be noted that heterogeneity may be under-estimated when only a small number of studies were available.

### Meta-analyses of device error frequency by sub-groups

The MAs described above were repeated by sub-group, i.e., i) diagnosis of asthma or COPD (asthma-only study, COPD-only study, mixed asthma/COPD study) and ii) previous device use (device-naïve patients, experienced device users, and a mix of naïve and experienced users).

### Meta-analyses for comparison of pairs of devices/pairs of device types

Data from all the studies that provided error frequencies for the same two devices (within the same study) were included in the MAs. Pairs of devices were compared using a REML random-effects MA provided that a minimum of five studies for each comparison was available.

### Meta-regression of device error frequency for each device/device type

Meta-regression is a tool for exploring the association of patient characteristics with outcomes of interest, thereby investigating sources of heterogeneity. Meta-regression aims to discern whether a relationship exists between an outcome measure and explanatory variables.

In order to assess the impact of baseline characteristics on the device error frequency, baseline characteristics reported in the studies were incorporated as regression factors into the REML random-effects MA model. Where available, the following baseline characteristics were considered for inclusion in the meta-regression analysis: i) population mean age; ii) proportion of current smokers; iii) proportion of males. However, as no significant findings were observed, these data are not reported.

### Sensitivity analysis

A sensitivity analysis was also conducted to assess any potential bias resulting from the inclusion of industry-sponsored studies. In this analysis, data for inhaler devices that were products of the pharmaceutical company sponsoring the clinical trial(s), were excluded from the meta-analyses. The results of the sensitivity analysis were compared to the original analysis where all relevant studies were included.

## Electronic supplementary material

Supplementary Information
