# Supplementary material for: Device errors in asthma and COPD: systematic literature review and meta-analysis
Source: NPJ Prim Care Respir Med. 2017 Apr 3;27:22. doi: 10.1038/s41533-017-0016-z (PMC5434773; doi:10.1038/s41533-017-0016-z)
Supplement: Supplementary file 1 — Supplementary Information [file 41533_2017_16_MOESM1_ESM.doc]

**Supplemental data**

**Device errors in COPD and asthma: systematic literature review and meta-analysis**

Henry Chrystyn, Job van der Palen, Raj Sharma, Neil Barnes, Bruno Delafont, Anadi Mahajan, and Mike Thomas

**Supplementary Table 1.** Search strategy

Embase/Medline search strategy

| **#** | **Query** | **Facet** | **Results, *n*** |
| --- | --- | --- | --- |
| 1 | 'inhaler'/exp | Device facet | 8,240 |
| 2 | 'metered dose inhaler'/exp |  | 4,512 |
| 3 | 'dry powder inhaler'/exp |  | 291 |
| 4 | 'inhalation'/exp |  | 17,447 |
| 5 | inhal*:ab,ti OR inhaler:ab,ti OR dpi:ab,ti OR mdi:ab,ti OR spacer:ab,ti OR turbuhaler:ab,ti OR turbohaler:ab,ti OR handihaler:ab,ti OR breezhaler:ab,ti OR aeroliser:ab,ti OR aerolizer:ab,ti OR autohaler:ab,ti OR respimat:ab,ti OR diskus:ab,ti OR accuhaler:ab,ti |  | 135,031 |
| 6 | #1 OR #2 OR #3 OR #4 OR #5 |  | 141,251 |
| 7 | error:ab,ti OR mishandling:ab,ti OR erroneous:ab,ti OR 'incorrect use':ab,ti OR 'incorrect technique':ab,ti OR 'improper use':ab,ti OR 'improper technique':ab,ti OR 'inadequate technique':ab,ti OR 'inadequate use':ab,ti OR 'insufficient use':ab,ti OR 'insufficient technique':ab,ti OR 'critical error':ab,ti OR 'significant error':ab,ti | Error facet | 147,486 |
| 8 | 'correct use':ab,ti OR 'correct technique':ab,ti OR 'proper use':ab,ti OR 'proper technique':ab,ti OR 'adequate technique':ab,ti OR 'adequate use':ab,ti OR 'sufficient use':ab,ti OR 'sufficient technique':ab,ti |  | 6,923 |
| 9 | #7 OR #8 |  | 154,524 |
| 10 | ‘asthma'/exp OR 'asthma':ab,ti | Disease facet | 224,535 |
| 11 | ‘chronic obstructive lung disease'/exp OR 'chronic obstructive lung disease':ab,ti OR 'copd' |  | 80,956 |
| 12 | #10 OR #11 |  | 288,858 |
| 13 | #6 AND #9 | Device and error facet | 1,180 |
| 14 | #9 AND #12 | Disease and error facet | 1,404 |
| 15 | #13 OR #14 | Combined | 2,099 |
| 16 | #13 OR #14 AND [english]/lim | Final | 1,851 |

Cochrane search strategy

| **#** | **Query** | **Facet** | **Results, *n*** |
| --- | --- | --- | --- |
| 1 | MeSH descriptor: [Nebulizers and Vaporizers] explode all trees | Device facet | 1,878 |
| 2 | MeSH descriptor: [Dry Powder Inhalers] explode all trees |  | 32 |
| 3 | MeSH descriptor: [Metered Dose Inhalers] explode all trees |  | 305 |
| 4 | MeSH descriptor: [Inhalation] explode all trees |  | 230 |
| 5 | inhaler or dpi or mdi or spacer or turbuhaler or turbohaler or handihaler or breezhaler or aeroliser or aerolizer or autohaler or respimat or diskus or accuhaler |  | 5,237 |
| 6 | #1 OR #2 OR #3 OR #4 OR #5 |  | 6,220 |
| 7 | error or mishandling or "incorrect use" or "incorrect technique" or "improper use" or "improper technique" or "inadequate technique" or "inadequate use" or "insufficient use" or "insufficient technique" or "critical error" or "significant error" | Error facet | 21,345 |
| 8 | correct use or "correct technique" or "proper use" or "proper technique" or "adequate technique" or "adequate use" or "sufficient use" or "sufficient technique" |  | 339 |
| 9 | #7 or #8 |  | 21,588 |
| 10 | MeSH descriptor: [Asthma] explode all trees | Disease facet | 9,331 |
| 11 | MeSH descriptor: [Pulmonary Disease, Chronic Obstructive] explode all trees |  | 2,567 |
| 12 | #10 or #11 |  | 11,763 |
| 13 | #6 and #9 | Device and error facet | 341 |
| 14 | #9 and #12 | Disease and error facet | 400 |
| 15 | #13 or #14 | Final | 591 |

MEDLINE® In-Process search strategy

| **#** | **Query** | **Facet** | **Results, *n*** |
| --- | --- | --- | --- |
| 1 | Search inhaler[Title/Abstract] | Device facet | 4,836 |
| 2 | Search ("dry powder inhaler" OR dpi[Title/Abstract]) |  | 4,529 |
| 3 | Search ("metered dose inhaler" or mdi[Title/Abstract]) |  | 3,767 |
| 4 | Search (((((((((((inhal*[Title/Abstract]) OR spacer[Title/Abstract]) OR turbuhaler[Title/Abstract]) OR handihaler[Title/Abstract]) OR breezhaler[Title/Abstract]) OR aeroliser[Title/Abstract]) OR aerolizer[Title/Abstract]) OR autohaler[Title/Abstract]) OR respimat[Title/Abstract]) OR diskus[Title/Abstract]) OR accuhaler[Title/Abstract]) |  | 103,146 |
| 5 | Search (#1 OR #2 OR #3 OR #4) |  | 108,055 |
| 6 | Search ((((((((((((((error[Title/Abstract]) OR mishandling[Title/Abstract]) OR erroneous[Title/Abstract]) OR "incorrect use"[Title/Abstract]) OR "incorrect technique"[Title/Abstract]) OR "improper use"[Title/Abstract]) OR "improper technique"[Title/Abstract]) OR "inadequate technique"[Title/Abstract]) OR "inadequate use"[Title/Abstract]) OR "insufficient use"[Title/Abstract]) OR "insufficient technique"[Title/Abstract]) OR "critical error"[Title/Abstract]) OR "significant error"[Title/Abstract])) | Error facet | 131,357 |
| 7 | Search (((((((("correct use"[Title/Abstract]) OR "correct technique"[Title/Abstract]) OR "proper use"[Title/Abstract]) OR "proper technique"[Title/Abstract]) OR "adequate technique"[Title/Abstract]) OR "adequate use"[Title/Abstract]) OR "sufficient use"[Title/Abstract]) OR "sufficient technique"[Title/Abstract]) |  | 5,235 |
| 8 | Search (#6 OR #7) |  | 136,382 |
| 9 | Search asthma[Title/Abstract] | Disease facet | 109,705 |
| 10 | Search (copd or "chronic obstructive pulmonary disease"[Title/Abstract]) |  | 56,096 |
| 11 | Search (#9 or #10) |  | 159,140 |
| 12 | Search (#5 AND #8) | Device and error facet | 859 |
| 13 | Search (#8 AND #11) | Disease and error facet | 791 |
| 14 | Search (#12 OR #13) | Combined | 1,372 |
| 15 | Search (#14 AND (inprocess​[sb] OR  pubstatusa​headofprin​t)) | Final | 77 |

**Supplementary Table 2. Characteristics of studies involving adult patients with asthma or COPD that reported overall or critical errors**

| **Study** | **Diagnosis** | **Setting** | **Study design** | **Type of inhaler (no. patients)** | **Quality rating (overall risk of bias assessment)** | **No. of checklist steps** | | **% patients with ≥1 error** | |
| --- | --- | --- | --- | --- | --- | --- | --- | --- | --- |
|  |  |  |  |  |  | **All** | **Critical** | **Overall** | **Critical** |
| Epstein, 1979*^1^ | AS/COPD | Outpatient | Cross-sectional | MDI (*n* = 130) | Moderate risk | 11 |  | 89.2 |  |
| Allen, 1986^2^ | AS/COPD | Outpatient | Cross-sectional | MDI (*n* = 30) | Moderate risk | 6 | 4 | 90 | 40 |
| Horsley, 1988^3^ | AS/COPD | Mixed | Prospective | MDI (*n* = 86) | Moderate risk | 9 |  | 95.3 (57.3) |  |
| Hilton, 1990^4^ | AS | Outpatient | Cross-sectional | MDI (*n* = 262) MDI with spacer (*n* = 36) Turbuhaler® (*n* = 23) | Moderate risk | 4 4 4 |  | 55 42 22 |  |
| Zainudin, 1990^5^ | AS | Outpatient | Cross-sectional | MDI (*n* = 93) | Moderate risk | 8 |  | 62.4 |  |
| Larsen, 1994*^6^ | AS/COPD | Outpatient | Cross-sectional | MDI (*n* = 501) | Low risk | 9 |  | 89.2 (77.5)^¶^ |  |
| Liard, 1995*^7^ | AS | Outpatient | Cross-sectional | MDI (*n* = 668) | Moderate risk | 5 |  | 77.9 |  |
| Shrestha, 1996^8^ | AS | Inpatient | Cross-sectional | MDI (*n* = 125) | Moderate risk | 7 |  | 79 |  |
| Erickson, 1998^9^ | AS/COPD | Outpatient | Cross-sectional | MDI (*n* = 159) | Moderate risk | 9 |  | 94.3 (95.6)^ǁ^ |  |
| Plaza, 1998*^10^ | AS/COPD | Outpatient | Cross-sectional | MDI (*n* = 746) | Moderate risk | 9 |  | 91 |  |
| Giraud, 2002*^11^ | AS | Outpatient | Cross-sectional | MDI (*n* = 3955) | Low risk | 12 |  | 71 |  |
| Giraud, 2011^12^ | AS | Outpatient | Prospective | Autohaler® (*n* = 6,387) | Low risk | 12 |  | 59.9 (8.6)^‡^ |  |
| Sarvis, 2004^13^ | AS | Outpatient | Prospective | MDI (*n* = 33) | Moderate risk | 3 |  | 93.9 (48.5)^‡^ |  |
| Al-Hassan, 2009^14^ | COPD | Outpatient | Cross-sectional | MDI (*n* = 100) | Moderate risk | 9 |  | 100 |  |
| Souza, 2009^15^ | COPD | Outpatient | Cross-sectional | MDI (*n* = 23) Aerolizer® (*n* = 54) | Moderate risk | 7 7 |  | 95.7 90.7 |  |
| Souza, 2009^15^ | AS | Outpatient | Cross-sectional | MDI (*n* = 44) Aerolizer® (*n* = 47) | Moderate risk | 7 7 |  | 95.5 87.2 |  |
| Hashmi, 2012^16^ | AS | Outpatient | Cross-sectional | MDI (*n* = 215) | Moderate risk | NR |  | 83.7 |  |
| Al-Jahdali, 2013^17^ | AS | Inpatient | Cross-sectional | MDI (*n* = 414) MDI with spacer (*n* = 19) Turbuhaler® (*n* = 13) Diskus® (*n* = 4) | Low risk | 6 8 5 6 |  | 45.7 31.9 4.2 50 |  |
| Vargas, 2013^18^ | AS/COPD | Inpatient | Cross-sectional | MDI (*n* = 191) | Moderate risk | 7 |  | 99.5 |  |
| Arora, 2014^19^ | AS/COPD | Outpatient | Cross-sectional | MDI (*n* = 70) MDI with spacer (*n* = 50) | Moderate risk | NR NR |  | 94.3 78 |  |
| Van der Palen, 1995^20^ | COPD | Outpatient | Cross-sectional | MDI (*n* = 25) | Moderate risk | 8 | 3 |  | 76 |
| Hesselink, 2001^21^ | AS/COPD | Outpatient | Cross-sectional | MDI (n = 40) Turbuhaler® (*n* = 102) | Moderate risk |  | 4 4 |  | 30 31.4 |
| Molimard, 2003^22^ | AS/COPD | Outpatient | Cross-sectional | MDI (*n* = 552)  Turbuhaler® (*n* = 868)  Diskus® (*n* = 894)  Aerolizer® (*n* = 769)  Autohaler® (*n* = 728) | Low risk | 7 6 6 6 5 | 3 4 3 4 3 | 76 54 49 54 55 | 28 32 11 12 11 |
| Ho, 2004^23^ | AS/COPD | Outpatient | Cross-sectional | MDI (*n* = 39)  MDI with spacer (*n* = 34) | Low risk | 13 13 | 8 8 |  | 17.9 2.9 |
| Melani, 2004^24^ | AS/COPD | Outpatient | Cross-sectional | MDI (*n* = 866)  MDI with spacer (*n* = 190)  Turbuhaler® (*n* = 524)  Diskus® (*n* = 475)  Aerolizer (*n* = 230) | Low risk |  | 4 7 4 4 4 |  | 23.9 2.6 23.1 23.9 16.8 |
| Khassawneh, 2008^25^ | AS/COPD | Outpatient | Cross-sectional | MDI (*n* = 193)  Turbuhaler® (*n* = 146)  Diskus® (*n* = 103)  Aerolizer (*n* = 83) | Moderate risk |  | 3 4 3 4 |  | 74.6 43.2 6.8 16.9 |
| Rootmensen, 2010^26^ | AS/COPD | Outpatient | Cross-sectional | MDI (*n* = 32)  MDI with spacer (*n* = 36)  Turbuhaler® (*n* = 51)  Diskus® (*n* = 41) | Low risk |  | 5 3 2 2 |  | 81 47 18 15 |
| Melani, 2011^27^ | AS/COPD | Outpatient | Cross-sectional | MDI (*n* = 843) Turbuhaler® (*n* = 361)  Diskus® (*n* = 467)  Handihaler (*n* = 587) | Moderate risk |  | 5 4 4 4 |  | 12 43.5 34.5 35 |
| Batterink, 2012^28^ | COPD | Inpatient | Cross-sectional | MDI (*n* = 14) Turbuhaler® (*n* = 10) Diskus® (*n* = 5) Handihaler® (*n* = 6) | Moderate risk | 8 7 8 9 | 4 4 4 4 |  | 93 50 20 50 |
| Connolly, 1995^§29^ | AS/COPD | Outpatient | Prospective | MDI (*n* = 40; 10) MDI with spacer (*n* = 40; 30) | Moderate risk | 7 6 | 4 3 | 60 32.5 | 30 (20)^‡^ 7.5 (3.3)^‡^ |
| Bosnic-Anticevich, 2010^30^ | AS/COPD | Outpatient | RCT | MDI (*n* = 52) | Low risk | 8 |  | 94 |  |
| Owens-Harrison, 1996^31^ | COPD | Inpatient | RCT | MDI (*n* = 87) | Moderate risk | 8 |  | 85.1 |  |
| De Blaquiere, 1989^32^ | AS/COPD | Outpatient | RCT | MDI (*n* = 27) | Moderate risk | 3 |  | 44.4 (44.9)^‡^ |  |
| Dahl, 2003^33^ | COPD | Outpatient | RCT | MDI (*n* = 139) Handihaler® (*n* = 139) | Moderate risk | 12 12 |  | 45.3 (56.8)^‡^ 40.3 (46)^‡^ |  |
| Lenney, 2000^34^ | AS/COPD | Outpatient | Cross-sectional | MDI (*n* = 100) Turbuhaler® (*n* = 100) Autohaler® (*n* = 100) | Moderate risk | 5 5 5 |  | 21 13 9 |  |
| Basheti, 2011 (Australian sub-study)^35^ | AS | Outpatient | Cross-sectional | Turbuhaler® (*n* = 42) Diskus® (*n* = 53) | Moderate risk | 9 9 | 4 3 |  | 71 43 |
|  |  |  |  |  |  |  |  |  |  |
| Basheti, 2011 (Jordanian sub-study)^35^ | AS | Outpatient | Cross-sectional | Turbuhaler® (*n* = 40) Diskus® (*n* = 51) | Moderate risk | 9 9 | 4 3 |  | 100 20 |
| Wieshammer, 2008^36^ | AS/COPD | Outpatient | Cross-sectional | Turbuhaler® (*n* = 109) Diskus® (*n* = 86) Aerolizer® (*n* = 22) Handihaler® (*n* = 32) | Moderate risk |  | 3 3 4 4 |  | 34.9 26.7 9.1 53.1 |
| Nimmo, 1993^37^ | AS/COPD | Inpatient | RCT | Turbuhaler® (*n* = 16) | High risk | 9 |  | 94 (19)^‡^ |  |
| Serra-Batlles, 2002^38^ | AS/COPD | Outpatient | RCT | Turbuhaler® (*n* = 169) Diskus® (*n* = 169) | Low risk | 7 5 |  | 82 (38)^‡^ 66 (28)^‡^ |  |
| Van der Palen, 1998*^§39^ | AS/COPD | Outpatient | RCT | Turbuhaler® (*n* = 50) Diskus® (*n* = 50) | Moderate risk | 10 9 | 4 3 | 54 50 | 26 8 |
| Wilson, 2007^§40^ | COPD | Outpatient | RCT | Turbuhaler® (*n* = 30)  Diskus® (*n* = 30)  Handihaler® (*n* = 30) | Moderate risk | 12  12  12 | 1  1  1 |  | 6.7 (6.7)^‡^ 3.3 (3.3)^‡^ 23.3 (30)^‡^ |
| Garcia-Cardenas, 2013 (Control, BL)^41^ | AS | Outpatient | RCT | Turbuhaler® (*n* = 150) | Low risk | 10 |  | 76 |  |
| Garcia-Cardenas, 2013 (Intervention, BL)^41^ | AS | Outpatient | RCT | Turbuhaler® (*n* = 186) | Low risk | 10 |  | 80.5 |  |
| Liam, 2000^42^ | AS | Outpatient | Prospective | Diskus® (*n* = 48) | Moderate risk | 4 |  | 33.3 |  |
| van der Palen, 2007*^§43^ | COPD | Outpatient | RCT | Diskus® (*n* = 60)  Handihaler® (*n* = 60) | Moderate risk | 8  13 |  | 68.3 (18.3)^‡^ 66.7 (13.3)^‡^ |  |
| van der Palen, 2013*^§44^ | AS | Outpatient | RCT | Diskus® (*n* = 31) Elpenhaler® (*n* = 31) | Low risk | 9  12 | 6  9 | 13 (0)^‡^  23 (6.5)^‡^ |  |
| van der Palen, 2013*^§44^ | COPD | Outpatient | RCT | Diskus® (*n* = 82) Elpenhaler® (*n* = 82) | Low risk | 9  12 | 6  9 | 18 (2.4)^‡^ | 40 (13.4)^‡^ |
| van der Palen, 2013*^§44^ | AS/COPD | Outpatient | RCT | Diskus® (*n* = 113) Elpenhaler® (*n* = 113) | Low risk | 9  12 | 6  9 |  | 16.8 (1.8)^‡^  35.4 (11.5)^‡^ |
| Schulte, 2008*^§45^ | AS/COPD | Outpatient | RCT | Diskus® (*n* = 72) | Moderate risk | 11 | 3 | 31.9 (9.7)^‡^ |  |
| van der Palen, 2013b*^§46^ | COPD | Outpatient | RCT | Handihaler® or Genuair®  (*n* = 105, both) | Low risk | 11 | 9 |  | 26.7 |
| Chapman, 2011^47^ | COPD | Outpatient | RCT | Breezhaler® or Handihaler® (*n* = 82) | Moderate risk | 19 | 2 |  | 30 (19)^‡^ |
| Epstein, 2001^48^ | AS | Outpatient | Cross-sectional | Turbuhaler® (*n* = 105) | Moderate risk | 14 |  | 88.7 |  |

AS = asthma, BL = baseline, COPD = chronic obstructive pulmonary disease, MDI = metered dose inhaler, NR = not reported, RCT = randomised controlled trial. *Pharmaceutical-sponsored studies which were excluded in the sensitivity analysis. ^¶^Maximum method and minimum method, respectively. ^ǁ^Observed error rate and self-reported error rate, respectively. ^‡^Before and after device instruction, respectively. ^§^Inhaler naïve. Quality rating was assessed using criteria published by the Cochrane Collaboration for RCTs and the Newcastle Ottawa scale for cross-sectional and prospective studies.

**Appendix 1**

**Reasons for study/data exclusions from the analyses**

***Title/abstract screening***

After removal of duplicate citations due to overlapping of databases, 2,394 citations were screened for eligibility on the basis of their titles and abstracts. Following screening, a total of 2,248 citations were excluded and 146 citations were selected for full-text screening. All excluded citations were coded with the reason of exclusion, as detailed below:

- Reviews (*n* = 969): Reviews, editorials and commentaries that did not focus on the project objective
- Disease (*n* = 509): Studies conducted in diseases other than asthma/COPD. Studies that included a minor proportion of patients with other diseases but were receiving inhaled medications were included at this stage
- Outcomes not of interest (*n* = 417): Studies that did not report any outcome pertinent to device errors were excluded using this code
- Animal/in-vitro studies (*n* = 293): Studies conducted in animals or in-vitro studies conducted in tissues/laboratory set-up were excluded using this code
- Study in professionals (*n* = 33): Some of the studies assessed device inhalation technique amongst healthcare professionals such as physicians, nurses, pharmacists, etc. The participants did not have any disease and were excluded using this code
- Language (*n* = 12): The review focused on only English language studies. Studies published in any language except English were excluded using this code
- Conference abstracts (*n* = 11): Studies published as conference abstracts are non-peer reviewed and provide very limited information and were excluded using this code
- Device not of interest (*n* = 4): Studies conducted in patients assessing devices not of interest (e.g. Diskhaler, Rotahaler etc.) were excluded using this code

***Full text screening***

Based upon title/abstract screening, full texts for the 146 included citations were retrieved. Amongst these, full texts for 13 studies could not be retrieved from either the journal homepages or the British library. The remaining 133 citations were screened, and a total of 73 studies* were included for extraction, based upon the eligibility screening. For the excluded studies, the reason for exclusion was coded, as detailed below:

- Conference abstracts (*n* = 20): Studies published as conference abstracts are non-peer reviewed and provide very limited information and were excluded using this code
- Children only (*n* = 19): The review focused on device errors in adolescents and adults; studies assessing inhalation technique in children (<12 years) were excluded using this code
- Outcomes not of interest (*n* = 7): Studies that did not report any outcome pertinent to device errors were excluded using this code
- Device not of interest (*n* = 5): Studies conducted in patients assessing devices not of interest (e.g. Diskhaler, Rotahaler etc.) were excluded using this code
- Reviews (*n* = 5): Reviews, editorials and commentaries that did not focus on the project objective were excluded using this code
- Study in professionals (*n* = 2): Some of the studies assessed device inhalation technique amongst healthcare professionals such as physicians, nurses, pharmacists, etc. The participants did not have any disease and were excluded using this code
- Duplicates (*n* = 2): Studies identified as duplicates of other included publications were excluded using this code

*A total of 72 primary studies were extracted from 73 publications. Amongst these, 40 studies were included in the quantitative analysis.

**Appendix 2**

**Additional results**

**Supplementary Figure 1**. Meta-analysis of overall error frequency for (a) Turbuhaler® and (b) Diskus® in prospective/cross-sectional studies

**
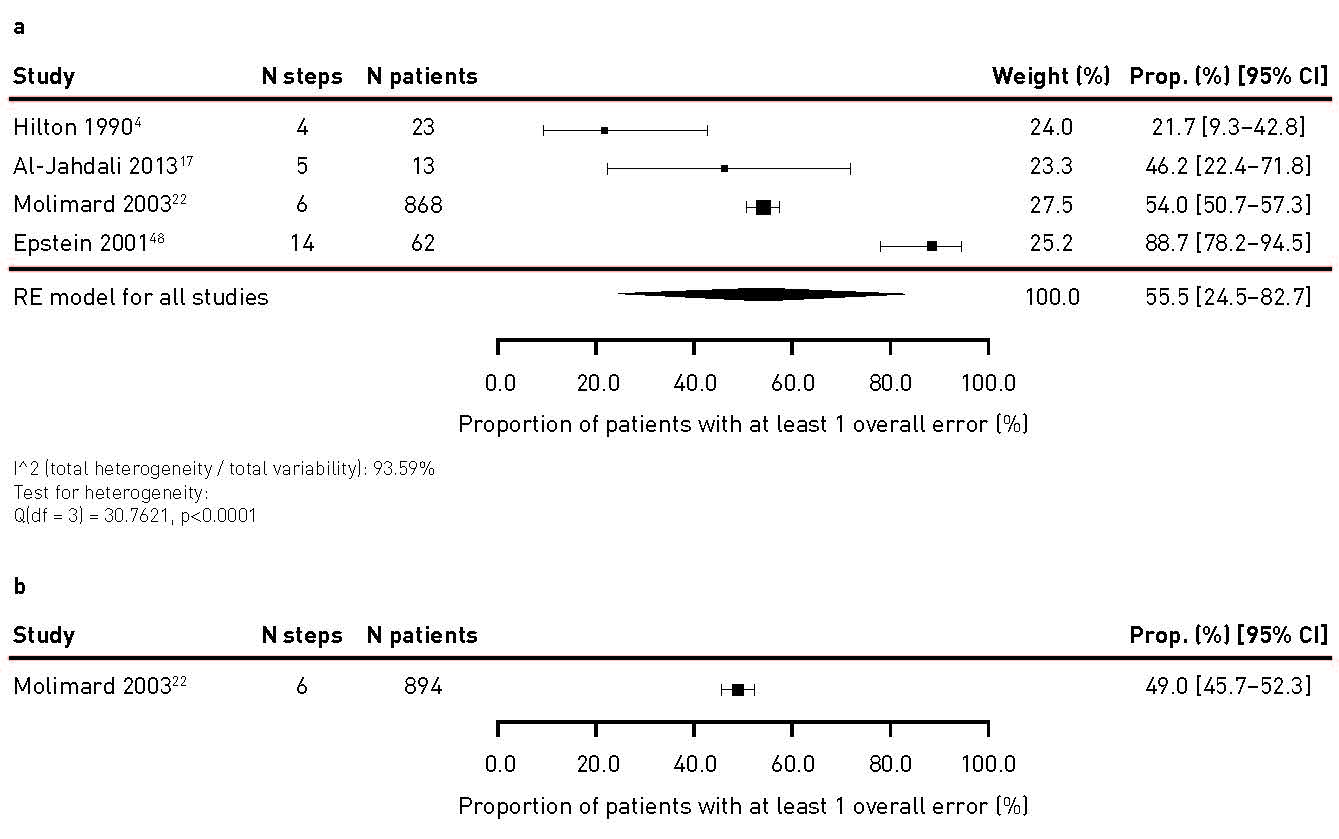
**

**Supplementary Figure 2.** Meta-analysis of critical error frequency for (a) Turbuhaler® and (b) Diskus® in prospective/cross-sectional studies

**
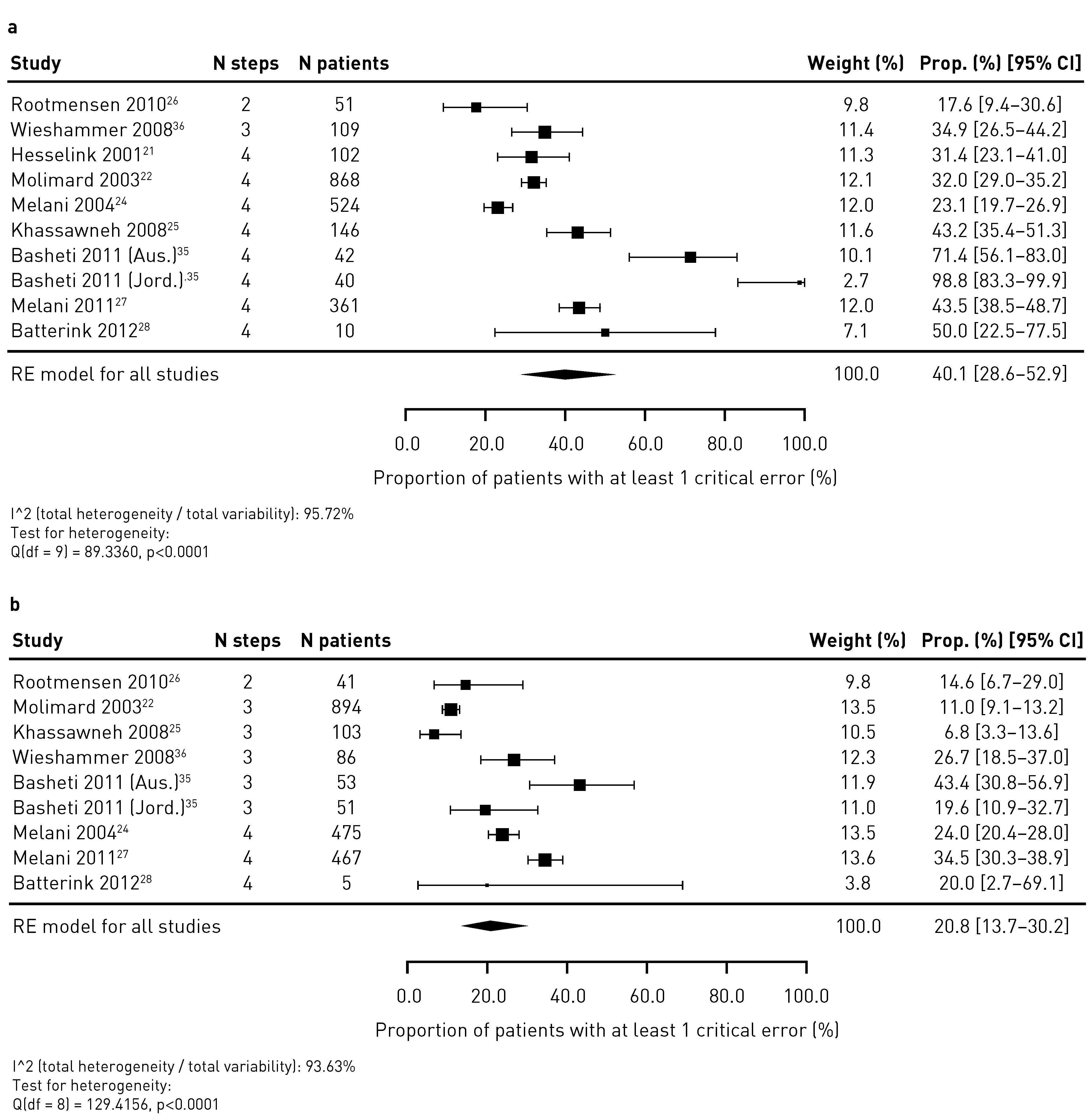
**

**Appendix 3**

**Sensitivity analysis removing industry-sponsored data**

A sensitivity analysis was conducted to assess any potential bias resulting from the inclusion of industry-sponsored studies. In this analysis, data for inhaler devices that were products of the pharmaceutical company sponsoring the clinical trial(s) were excluded from the meta-analyses. Data were excluded from seven studies.^29,39,40,43–46^ The results of the sensitivity analysis were compared to the original analysis where all relevant studies were included. The sensitivity analysis provided similar results (see Supplementary Figures 3–6). There were insufficient studies available to conduct the sensitivity analysis of overall errors for Turbuhaler® and Diskus®.

**Supplementary Figure 3.** Meta-analysis of overall error frequency for MDIs in prospective/cross-sectional studies - sensitivity analysis


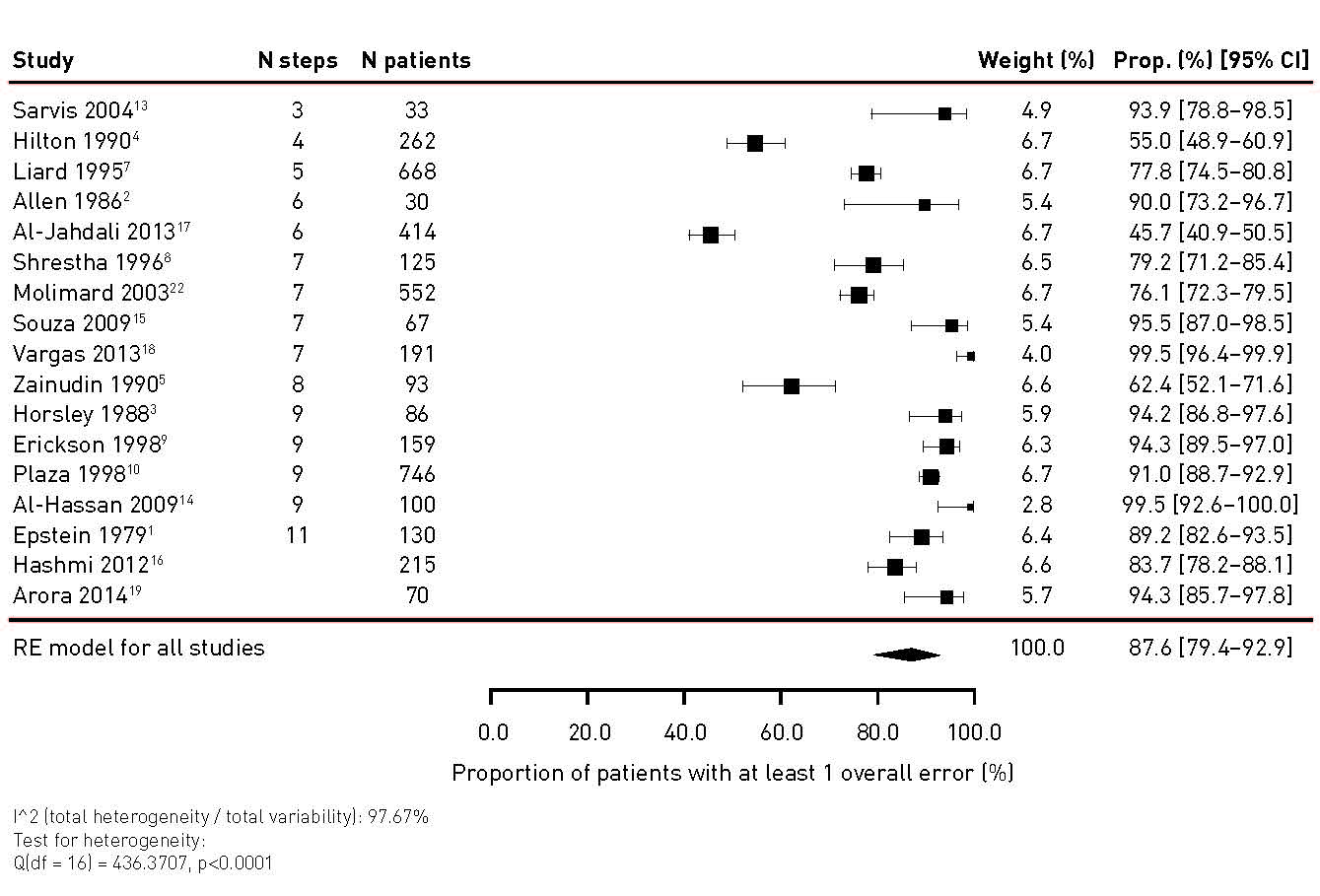


**Supplementary Figure 4.** Meta-analysis of critical error frequency for MDIs in prospective/cross-sectional studies - sensitivity analysis


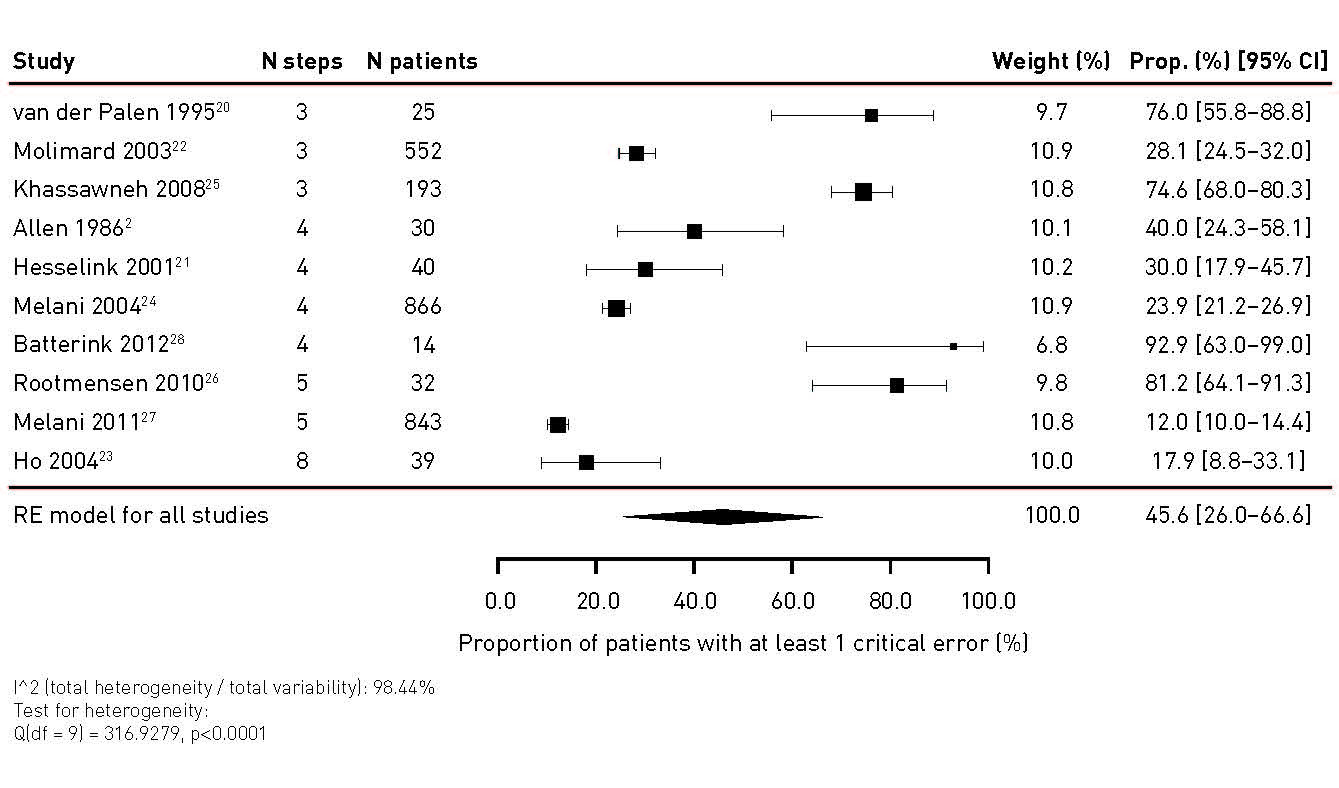


**Supplementary Figure 5.** Meta-analysis of critical error frequency for Turbuhaler® in prospective/cross-sectional studies - sensitivity analysis


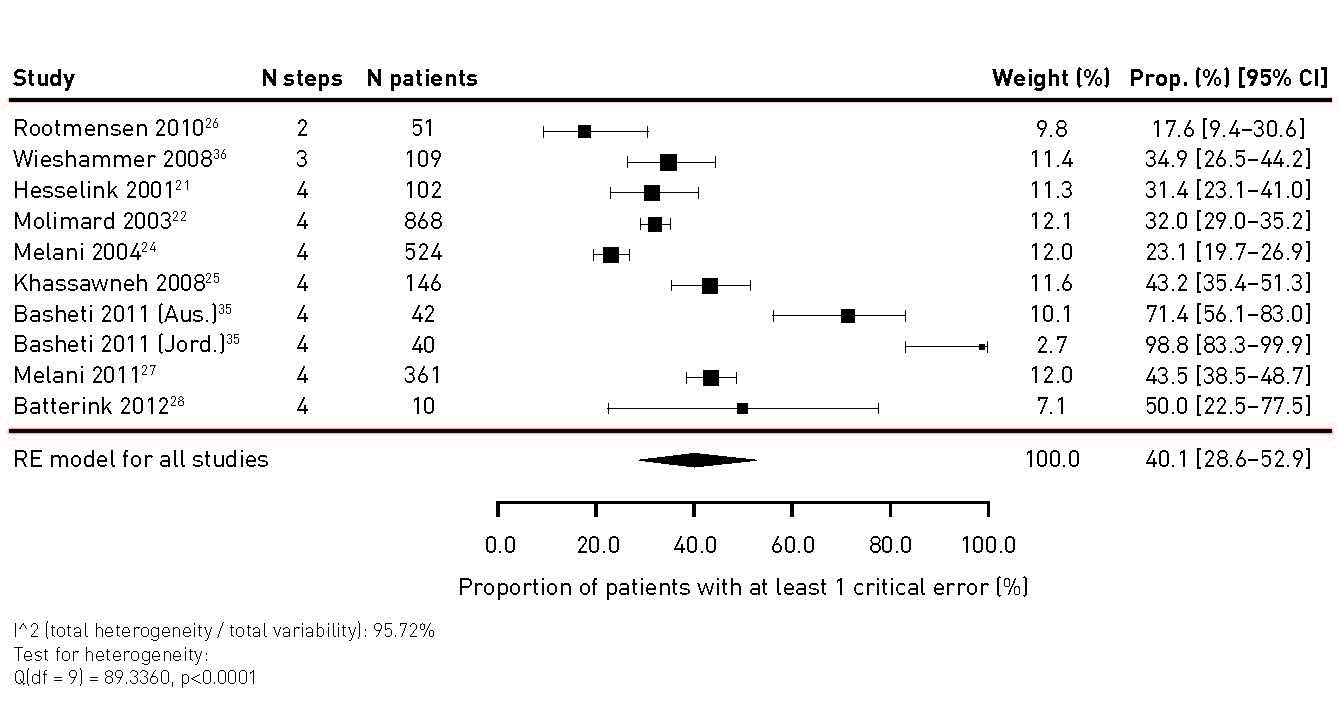


**Supplementary Figure 6.** Meta-analysis of critical error frequency for Diskus® in prospective/cross-sectional studies - sensitivity analysis

**
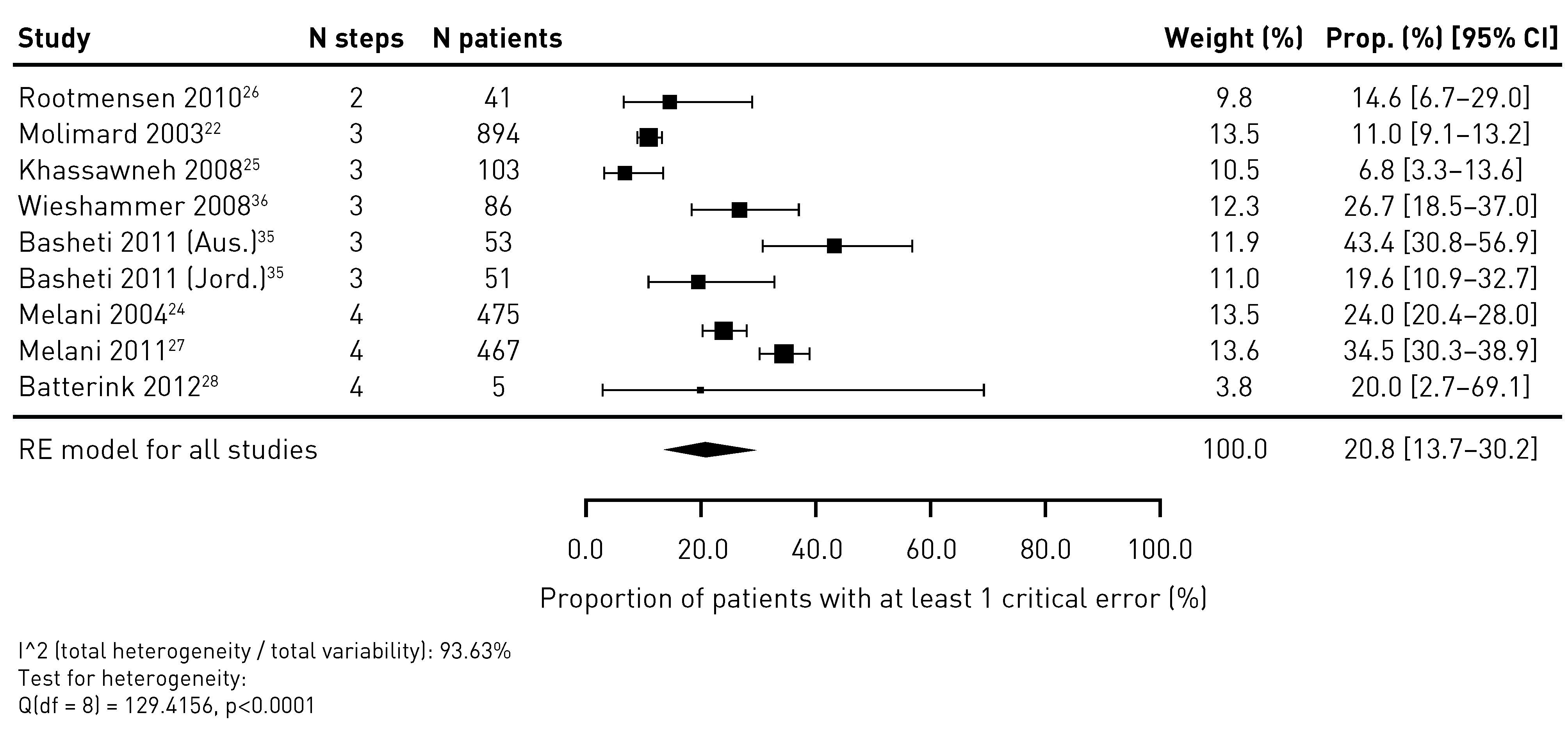
**

**References**

1. Epstein SW, Manning CPR, Ashley MJ, Corey PN. Survey of the clinical use of pressurized aerosol inhalers. *Can Med Assoc J*. **120**, 813–6 (1979)
2. Allen SC, Prior A. What determines whether an elderly patient can use a metered dose inhaler correctly? *Br J Dis. Chest.* **80**, 45–9 (1986)
3. Horsley MG, Bailie GR. Risk factors for inadequate use of pressurized aerosol inhalers. *J Clin Pharm Ther*. **13**, 139–43 (1988)
4. Hilton S. An audit of inhaler technique among asthma patients of 34 general practitioners. *Br J Gen Pract*. **40**, 505–6 (1990)
5. Zainudin BM, Sufarlan AW. Incorrect use of pressurised metered dose inhaler by asthmatic patients. *Med J Malaysia*. **45**, 235–8 (1990)
6. Larsen JS, Hahn M, Ekholm B, Wick KA. Evaluation of conventional press-and-breathe metered-dose inhaler technique in 501 patients. *J Asthma***. 31**, 193–9 (1994)
7. Liard R, Zureik M, Aubier M, Korobaeff M, Henry C, Neukirch F. Misuse of pressurized metered dose inhalers by asthmatic patients treated in French private practice. *Rev Epidemiol Sante Publ.* **43**, 242–9 (1995)
8. Shrestha M, Parupia H, Andrews B, Kim SW, Martin MS, Park DI, et al. Metered-dose inhaler technique of patients in an urban ED: Prevalence of incorrect technique and attempt at education. *Am J Emerg Med*. **14**, 380–4 (1996)
9. Erickson SR, Horton A, Kirking DM. Assessing metered-dose inhaler technique: Comparison of observation vs. patient self-report. *J Asthma* **35**, 575–83 (1998)
10. Plaza V, Sanchis J. Medical personnel and patient skill in the use of metered dose inhalers: A multicentric study. *Respiration.* **65**, 195–8 (1998)
11. Giraud V Roche N. Misuse of corticosteroid metered-dose inhaler is associated with decreased asthma stability. *Eur Respir J.* **19**, 246–51 (2002)
12. Giraud V, Allaert FA, Magnan A. A prospective observational study of patient training in use of the autohaler (registered trademark) inhaler device: The Sirocco Study. *Eur Rev Med Pharmacol Sci*. **15**, 563–70 (2011)
13. Sarvis CA, Tice AL, Plake KS. Evaluation of inhaler technique using the aerosol inhalation monitor. *J Pharm Pract.* **17**, 275–9 (2004)
14. Al-Hassan MI. Assessment of inhaler technique in patients attending a chest hospital in Riyadh City. *Int J Pharmacol.* **5**, 232–5 (2009)
15. Souza ML, Meneghini AC, Ferraz E, Vianna EO, Borges MC. Knowledge of and technique for using inhalation devices among asthma patients and COPD patients. *J Bras Pneumol*. **35**, 824–31 (2009)
16. Hashmi A, Soomro JA, Memon A, Soomro TK. Incorrect inhaler technique compromising quality of life of asthmatic patients. *J Med.* **13**, 16–21 (2012).
17. Al-Jahdali H, Ahmed A, Al-Harbi A, Khan M, Baharoon S, Bin Salih S, et al. Improper inhaler technique is associated with poor asthma control and frequent emergency department visits. *Allergy Asthma Clin Immunol*. **9**, 8 (2013)
18. Vargas O, Martinez J, Ibanez M, Pena C, Santamaria M. The use of metered-dose inhalers in hospital environments. *J Aerosol Med Pulm Drug Deliv*. **26**, 287–96 (2013)
19. Arora P, Kumar L, Vohra V, Sarin R, Jaiswal A, Puri MM, et al. Evaluating the technique of using inhalation device in COPD and Bronchial Asthma patients. *Respir Med*. **108**, 992–8 (2014)
20. van der Palen J, Klein JJ, Kerkhoff AH, van Herwaarden CL. Evaluation of the effectiveness of four different inhalers in patients with chronic obstructive pulmonary disease. *Thorax.* **50**, 1183–7 (1995)
21. Hesselink AE, Penninx BW, Wijnhoven HA, Kriegsman DM, van Eijk JT. Determinants of an incorrect inhalation technique in patients with asthma or COPD. *Scand J Prim Health Care.* **19**, 255–60 (2001)
22. Molimard M, Raherison C, Lignot S, Depont F, Abouelfath A, Moore N. Assessment of handling of inhaler devices in real life: An observational study in 3811 patients in primary care. *J Aerosol Med Deposition Clear Eff Lung.* **16**, 249–54 (2003)
23. Ho SF, O’Mahony MS, Steward JA, Breay P, Burr ML. Inhaler technique in older people in the community. *Age Ageing.* **33**, 185–8 (2004)
24. Melani AS, Zanchetta D, Barbato N, Sestini P, Cinti C, Canessa PA, et al. Inhalation technique and variables associated with misuse of conventional metered-dose inhalers and newer dry powder inhalers in experienced adults. *Ann Allergy Asthma Immunol.* **93**, 439–46 (2004)
25. Khassawneh BY, Al-Ali MK, Alzoubi KH, Batarseh MZ, Al-Safi SA, Sharara AM, et al. Handling of inhaler devices in actual pulmonary practice: Metered-dose inhaler versus dry powder inhalers. *Respir Care.* **53**, 324–8 (2008)
26. Rootmensen GN, Van Keimpema ARJ, Jansen HM, de Haan RJ. Predictors of incorrect inhalation technique in patients with asthma or COPD: A study using a validated videotaped scoring method. *J Aerosol Med Pulm Drug Deliv.* **23**, 323–8 (2010)
27. Melani AS, Bonavia M, Cilenti V, Cinti C, Lodi M, Martucci P et al. Inhaler mishandling remains common in real life and is associated with reduced disease control. *Respir Med.* **105**, 930–8 (2011)
28. Batterink J, Dahri K, Aulakh A, Rempel C*.* Evaluation of the use of inhaled medications by hospital in patients with chronic obstructive pulmonary disease. *Can J Hosp Pharm.* **65**, 111–8 (2012)
29. Connolly MJ. Inhaler technique of elderly patients: Comparison of metered-dose inhalers and large volume spacer devices. *Age Ageing* **24**, 190–2 (1995)
30. Bosnic-Anticevich SZ, Sinha H, So S, Reddel HK. Metered-dose inhaler technique: The effect of two educational interventions delivered in community pharmacy over time. *J Asthma* **47**, 251–6 (2010)
31. Owens-Harrison G, Grimm R, Gray D, Harrison O. Evaluation of education provided by a pharmacist to hospitalized patients who use metered-dose inhalers. *Hosp Pharm.* **31**, 677–81 (1996)
32. De Blaquiere P, Christensen DB, Carter WB, Martin TR. Use and misuse of metered-dose inhalers by patients with chronic lung disease: A controlled, randomized trial of two instruction methods. *Am Rev Respir Dis*.**140**, 910–6 (1989)
33. Dahl R, Backer V, Ollgaard B, Gerken F, Kesten S. Assessment of patient performance of the HandiHaler(registered trademark) compared with the metered dose inhaler four weeks after instruction. *Respir Med.* **97**, 1126–33 (2003)
34. Lenney J, Innes JA, Crompton GK. Inappropriate inhaler use: assessment of use and patient preference of seven inhalation devices. EDICI. *Respir Med.* **94,** 496–500 (2000)
35. Basheti IA, Qunaibi E, Bosnic-Anticevich SZ, et al. User error with diskus and turbuhaler by asthma patients and pharmacists in Jordan and Australia. *Respir. Care* **56**, 1916–23 (2011)
36. Wieshammer S, Dreyhaupt J. Dry powder inhalers: Which factors determine the frequency of handling errors? *Respiration.* **75**, 18–25 (2008)
37. Nimmo CJR, Chen DNM, Martinusen SM, Ustad TL, Ostrow DN. Assessment of patient acceptance and inhalation technique of a pressurized aerosol inhaler and two breath-actuated devices. *Ann Pharmacother*. **27**, 922–7 (1993)
38. Serra-Batlles J, Plaza V, Badiola C, Morejón E; Inhalation Devices Study Group. Patient perception and acceptability of multidose dry powder inhalers: A randomized crossover comparison of Diskus/Accuhaler with Turbuhaler. *J Aerosol Med Deposition Clear Eff Lung.* **15**, 59–64 (2002)
39. van der Palen J, Klein JJ, Schildkamp AM. Comparison of a new multidose powder inhaler (Diskus/Accuhaler) and the Turbuhaler regarding preference and ease of use. *J Asthma.* **35**,147–52 (1998)
40. Wilson DS, Gillion MS, Rees PJ. Use of dry powder inhalers in COPD. *Int J Clin Pract*. **61**, 2005–8 (2007)
41. Garcia-Cardenas V, Sabater-Hernandez D, Kenny P, Martínez-Martínez F, Faus MJ, Benrimoj SI. Effect of a pharmacist intervention on asthma control. A cluster randomized trial. *Respir Med*. **107**, 1346–55 (2013)
42. Liam C-K, Lim K-H, Wong CMM. Acceptance of the Accuhaler, a multidose powder inhaler, among asthmatic patients: A comparison with the pressurized metered-dose inhaler. *Asian Pac J Allergy Immunol*.**18**, 135–40 (2000)
43. van der Palen J, Eijsvogel ME, Kuipers BF, Schipper M, Vermue NA. Comparison of the Diskus® Inhaler and the Handihaler® Regarding Preference and Ease of Use. *J Aerosol Med*.**20**, 38–44 (2007)
44. van der Palen J, van der Valk P, Goosens M, Groothuis-Oudshoorn K, Brusse-Keizer M. A randomized cross-over trial investigating the ease of use and preference of two dry powder inhalers in patients with asthma or chronic obstructive pulmonary disease. *Expert Opin Drug Deliv*. **10**, 1171–8 (2013)
45. Schulte M, Osseiran K, Betz R, Wencker M, Brand P, Meyer T, et al. Handling of and preferences for available dry powder inhaler systems by patients with asthma and COPD. *J Aerosol Med Pulm Drug Deliv.* **21**, 321–8 (2008)
46. van der Palen J, [Ginko T](http://www.ncbi.nlm.nih.gov/pubmed/?term=Ginko T%5BAuthor%5D&cauthor=true&cauthor_uid=23745954), [Kroker A](http://www.ncbi.nlm.nih.gov/pubmed/?term=Kroker A%5BAuthor%5D&cauthor=true&cauthor_uid=23745954), [van der Valk P](http://www.ncbi.nlm.nih.gov/pubmed/?term=van der Valk P%5BAuthor%5D&cauthor=true&cauthor_uid=23745954), [Goosens M](http://www.ncbi.nlm.nih.gov/pubmed/?term=Goosens M%5BAuthor%5D&cauthor=true&cauthor_uid=23745954), [Padullés L](http://www.ncbi.nlm.nih.gov/pubmed/?term=Padullés L%5BAuthor%5D&cauthor=true&cauthor_uid=23745954), et al. Preference, satisfaction and errors with two dry powder inhalers in patients with COPD. *Expert Opin Drug Deliv*.**10**, 1023–31 (2013b)
47. Chapman KR, [Fogarty CM](http://www.ncbi.nlm.nih.gov/pubmed/?term=Fogarty CM%5BAuthor%5D&cauthor=true&cauthor_uid=21760722), [Peckitt C](http://www.ncbi.nlm.nih.gov/pubmed/?term=Peckitt C%5BAuthor%5D&cauthor=true&cauthor_uid=21760722), [Lassen C](http://www.ncbi.nlm.nih.gov/pubmed/?term=Lassen C%5BAuthor%5D&cauthor=true&cauthor_uid=21760722), [Jadayel D](http://www.ncbi.nlm.nih.gov/pubmed/?term=Jadayel D%5BAuthor%5D&cauthor=true&cauthor_uid=21760722), [Dederichs J](http://www.ncbi.nlm.nih.gov/pubmed/?term=Dederichs J%5BAuthor%5D&cauthor=true&cauthor_uid=21760722), et al. Delivery characteristics and patients' handling of two single-dose dry-powder inhalers used in COPD. *Int J Chron Obstruct Pulmon Dis.* **6**, 353–63 (2011)
48. Epstein S, Maidenberg A, Hallett D, Khan K, Chapman KR. Patient handling of a dry-powder inhaler in clinical practice. *Chest.* **120**, 1480–4 (2001)
